# Incidence, demographics, and survival of malignant hemangioendothelioma in the United States

**DOI:** 10.1002/cam4.6181

**Published:** 2023-06-01

**Authors:** Kelly G. Paulson, Vinod Ravi, Brian P. Rubin, Min Park, Elizabeth T. Loggers, Lee D. Cranmer, Michael J. Wagner

**Affiliations:** ^1^ Medical Oncology Providence‐Swedish Cancer Institute Seattle Washington USA; ^2^ Sarcoma Medical Oncology MD Anderson Cancer Center Houston Texas USA; ^3^ Robert J. Tomsich Pathology and Laboratory Medicine Institute, Cleveland Clinic Cleveland Ohio USA; ^4^ Clinical Research Division Fred Hutchinson Cancer Center Seattle Washington USA; ^5^ Division of Medical Oncology University of Washington Seattle Washington USA

**Keywords:** EHE, epithelioid hemangioendothelioma, hemangioendothelioma, rare cancer, sarcoma, SEER, USCS

## Abstract

**Background:**

Malignant hemangioendothelioma is an endothelial cancer with heterogeneous clinical behavior that can range from indolent to aggressive, of which the majority are epithelioid (EHE). Its incidence and demographics have not been previously well defined in a large cohort.

**Methods:**

This retrospective analysis used the US Cancer Statistics National Program of Cancer Registries – Surveillance Epidemiology End Results (SEER) combined database to identify patients in the US newly diagnosed with hemangioendothelioma between the years of 2001 and 2017 (*n* = 1986). Survival analyses were performed on a subset of patients within the SEER‐18 database with survival information available (*n* = 417). Outcomes included incidence, demographics of patients newly diagnosed with hemangioendothelioma, extent of disease at presentation, and overall survival.

**Results:**

The incidence of hemangioendothelioma in the US is 0.4 cases per million person‐years. Although cases rose to 122 newly diagnosed in the year 2017 (90 EHE, 32 other hemangioendothelioma), incidence rates were stable. Skin and connective tissues were the most common presenting sites (33.4%), followed by liver (24.5%), lung (17.6%), and bone (12.5%). Median age at diagnosis was 55 years; 27.2% of patients were pediatric, adolescent, or young adult (<40 years). At presentation, 36.4% of patients had localized disease; 21.6% presented with regional and 41.7% with distant metastases. Observed survival at 3 years was 79.7%, 70.7%, and 46.0% for patients presenting with local, regional, and distant disease and most deaths occurred within the first 2 years.

**Conclusions:**

Malignant hemangioendothelioma is ultra‐rare but meaningfully impacts affected patients. These data may provide benchmarks for comparison of new approaches to hemangioendothelioma therapy and highlight poor survival outcomes.

## Graphical abstract

Hemangioendothelioma is ultra‐rare with incidence of 0.4 cases per million person‐years but meaningfully impacts affected patients. These data may provide benchmarks for comparison of new approaches to hemangioendothelioma therapy and highlight poor survival outcomes.

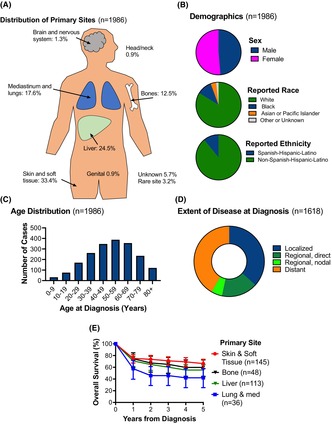

## 1 INTRODUCTION

Malignant hemangioendotheliomas are ultra‐rare vascular sarcomas with variable clinical behavior that can be either indolent or aggressive; the majority of malignant hemangioendotheliomas are epithelioid (EHE). The overall incidence has previously been reported as 0.38 per 1,000,000 person‐years in a large European database,^1^ but has been reported to be only 0.23 per 1,000,000 person‐years in the United States (US).^2^ Most (83%) are molecularly characterized by the presence of a *WWTR1‐CAMTA1* gene fusion and have a 5‐year overall survival (OS) of 59% in case series.^3^, ^4^, ^5^ A smaller subset has a *YAP1‐TFE3* gene fusion, associated with better 5‐year survival of 86%.^5^, ^6^, ^7^

Due to the extreme rarity of EHE, the patterns of initial presentation have not been well characterized in a large cohort. A consensus statement of EHE experts emphasized the need for data to guide surveillance strategies and to establish clinical benchmarks for future clinical trials.^8^ Herein, we report incidence, clinical presentation, and survival outcomes for US patients with malignant hemangioendothelioma.

## 2 METHODS

### 2.1 Population and data

IRB approval was obtained (Providence 202000564) and this research was conducted in accordance with Helsinki principles. De‐identified aggregate national registry data was extracted from public use databases of the US Cancer Statistics(USCS) National Program of Cancer Registries – Surveillance Epidemiology End Results (SEER) submission for the years 2001–2017.^9^ Data met high‐quality data criteria.^10^ All cases from years 2001 to 2017 with the ICD‐O‐3 codes 9130/3 (hemangioendothelioma) and 9133/3 (EHE) were included. Of note, these codes include only malignant hemangioendotheliomas, subtypes such as retiform haemangioendothelioma (9136/1), papillary intralymphatic angioendothelioma (9135/1), composite haemangioendothelioma (9136/1), pseudomyogenic haemangioendothelioma (9138/1) and kaposiform haemangioendothelioma have “benign” ICD‐O‐3 codes and are thus not included in national registry data. For cases diagnosed from 2003 to 2017, 100% of the population is covered for all 50 U.S. states and the District of Columbia. In 2001 and 2002, cases that were diagnosed in Mississippi are not available, so 99% of the U.S. population is covered. In keeping with privacy requirements, case numbers <16 cases are suppressed. Survival data were available on the subset of cases both contained within SEER 18 databases and who had hemangioendothelioma diagnosed as their first malignancy.^11^

### 2.2 Statistical analyses

*p*‐value for significance was set a priori at 0.05. For incidence rate calculations, data were age‐adjusted to the 2000 US standard population (19 age groups; census P25–1130). Confidence interval (CI) estimation was performed using the methods of Tiwari et al.^12^ Trends were calculated using 2‐year averages with annual percentage change calculated by the weighted least squares method. Survival analyses were performed with actuarial methods at 1,2,3,4, and 5 years post‐diagnosis with 95% CIs. Relative survival was calculated with the Ederer II method^13^ and expected survival tables included age, sex, socioeconomic status, geography, and race.

### 2.3 Software

Data were extracted using SEER*Stat software version 8.4.0.1 released May 16, 2022.^14^ Statistical analyses were performed within SEER*Stat. Graphs were generated using GraphPad Prism version 9 (Dotmatics).

## 3 RESULTS

### 3.1 Incidence of malignant hemangioendothelioma

**FIGURE 1:**
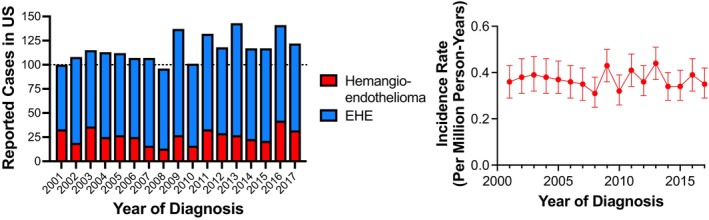
Newly diagnosed cases of malignant hemangioendothelioma in the United States, years 2001–2017. Incident cases recorded in the US Cancer Statistics (encompasses 99%–100% of newly diagnosed US cancers) increased from 100 cases in 2001 to 122 cases in 2017. Left panel: Incident cases. Right panel: Incidence rates. Data are presented +/− 95% CI.

Between 2001 and 2017, a total of 1986 cases of hemangioendothelioma were reported in the US (1542 EHE; 444 hemangioendothelioma). New cases increased from 100 in the year 2001 to 122 in 2017 (Figure 1). The adjusted incidence rate was ~0.4 cases per million person‐years across the study period (range 0.31–0.44 cases per million person‐years) and were statistically stable (annual percentage change −0.04%; 95% CI −1.06%–0.99%; Figure 1).

### 3.2 Site of malignant hemangioendothelioma presentation

**FIGURE 2:**
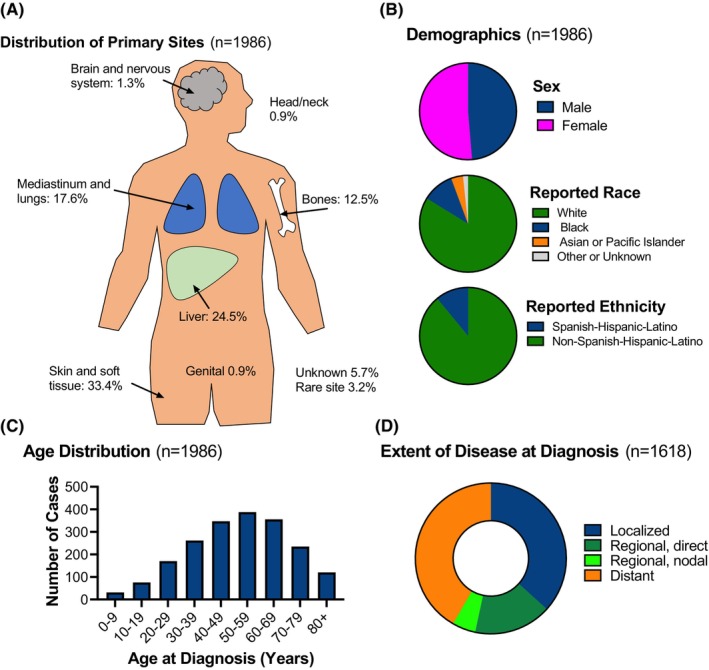
Characteristics and demographics of newly diagnosed malignant hemangioendotheliomas. Panels (A–C) include all 1986 hemangioendothelioma cases diagnosed in the US between years 2001 and 2017; panel (D) includes the 1618 cases with staging information. (A) Distribution of primary sites, (B) demographics, (C) age at diagnosis, and (D) extent of disease at diagnosis.

Characteristics of malignant hemangioendotheliomas given the diagnosis code 9130/3 (hemangioendothelioma) and 9133/3 (EHE) were similar; therefore, the populations were kept together for primary analysis. The most common sites of presentation were in the connective tissues/subcutaneous tissues/skin (33.4%; Figure 2A). This was followed by presentation in the liver (24.5%), mediastinum/lungs/pleura (17.6%), and bones (12.5%). Rare sites included brain (1.3%), head/neck (0.9%), genitalia (0.9%) and other. 114 cases (5.7%) had an unknown or unreported primary site.

### 3.3 Demographics

Among persons newly diagnosed with malignant hemangioendothelioma, 51.3% were female (48.7% male) while 83.3% were white, 10.5% Black, 3.9% Asian, and the remaining persons were unknown/unspecified, or in a racial group containing fewer than 16 cases (Figure 2B). 11.0% of patients were reported as Hispanic. Median age at diagnosis was 55 years (Figure 2C) and 27.2% of patients were pediatric, adolescent, or young adult (under 40 years). Figure S1 includes data for EHE alone.

### 3.4 Extent of disease at presentation

Local‐regional‐distant (LRD) staging information was available for 1618 of the 1986 patients (81.5%). 41.7% presented with distant metastatic disease while 21.6% of patients presented with regional disease, including 269 patients by direct extension alone, 42 patients by regional lymph nodes alone, and 39 patients regional by both modes. Approximately one‐third of patients (36.7%) presented with localized disease (Figure 2D).

### 3.5 Survival

**FIGURE 3:**
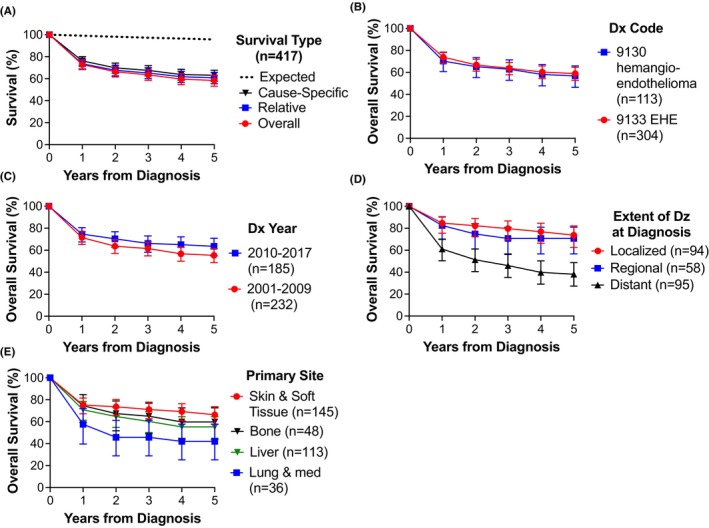
Survival after hemangioendothelioma diagnosis. Survival data were available for 417 patients with malignant hemangioendothelioma who were included in the SEER‐18 databases subset of the US Cancer Statistics. (A) Observed overall survival (OS) (red circles) as compared to expected survival based on demographic data (dotted line). Calculated relative survival (blue squares) and cause‐specific survival (black triangles) are also shown. (B) OS by diagnosis code (hemangioendothelioma or epithelioid hemangioendothelioma). (C) Survival by decade of diagnosis. Data are presented +/− 95% confidence interval. (D) OS by extent of disease at diagnosis. Survival if distant disease at diagnosis was significantly worsened from localized or regional disease (*p* < 0.05). (E) OS by reported primary site. Differences between lung/mediastinum and skin/soft tissue were significant (*p* < 0.05).

Survival information was available for a subset of 417 out of 1986 patients captured within SEER‐18 database (Section 2). Observed OS at 1, 3, and 5 years from diagnosis was 72.9% (95% CI 68.3%–77%), 63.5% (95% CI 58.5%–68.1%), and 58.4% respectively (95% CI 53.2%–63.2%; Figure 3A). This was substantially lower than expected survival based on age and demographic data (3‐year expected survival 97.3%). Relative survival (survival compared to expected) and disease‐specific survival were nearly identical to OS (3‐year relative survival 65.2% and cause‐specific survival 67.5%; Figure 3A). More than 80% of deaths occurred within the first 2 years after diagnosis. Survival by diagnosis code was identical (Figure 3B). A non‐significant trend was observed for improved survival with hemangioendothelioma diagnosis in the 2010s as compared to the 2000s (Figure 3C). OS at 3 years was 79.7%, 70.7%, and 46.0% for patients with local, regional, and distant disease respectively (Figure 3D; *n* = 247; survival significantly worsened for distant presentation). Patients with primary presentation in soft tissue/skin/subcutaneous had significantly improved outcomes (3‐year OS 71.1%; 95% CI 62.7%–77.9%; *n* = 145) versus those presenting with lung/mediastinal disease (Figure 3E; 45.8%; 95% CI 28.9%–61.2%; *p* < 0.05; *n* = 36); outcomes for liver (3‐year survival 60.2%; *n* = 113) or bone (3 year OS 65%; *n* = 48) were intermediate.

## 4 DISCUSSION

Hemangioendothelioma is an ultra‐rare sarcoma with a paucity of data to guide optimal treatment and counseling of patients. To our knowledge this is the largest cohort of malignant hemangioendothelioma/EHE patients reported to date. The US incidence rate in this series (120 new cases diagnosed in the US per year) is similar to that recently reported in France and approximately double the previously reported US incidence.^1^, ^2^ Prior differences likely reflected ascertainment bias, with earlier US data under‐representing the true incidence of EHE. We also found equal numbers of cases in men and women, whereas prior reports have suggested a female preponderance.^15^

Although EHE is often considered an indolent cancer, we observed 3‐year OS of only 63.5% which was far worse than expected survival based on actuarial data matched on sociodemographic and socioeconomic factors. This, combined with the fact that most deaths occurred within 5 years from diagnosis and most of those within the first 2 years, suggests that EHE is the major factor driving mortality and further supports the use of 1‐ or 3‐year OS with comparison to historical data as a feasible and clinically appropriate clinical trial endpoint. As expected, outcomes in patients with advanced disease at presentation were especially poor. This is consistent with prior reports identifying pulmonary infiltrates, pleural effusion, and ascites as negative prognostic signs in EHE.^15^ Indeed, in a recent prospective clinical trial for progressing EHE less than half of patients were still on study treatment at 6 months and 17% still on study treatment after 1 year.^16^ However, even patients presenting with localized disease had mortality of one in five, highlighting the ongoing need for more research and better therapies.

A limitation of our data set is that considering the rare nature of this tumor, misclassification bias is possible and genomic or biomarker data to confirm the diagnosis were not available in this national population‐based registry. Other types of hemangioendothelioma such as those with other gene fusions are likely also included in the “hemangioendothelioma” group. A second limitation is that the USCS registry only includes malignant tumors. Several types of hemangioendothelioma, including those with aggressive potential such as kaposiform hemangioendothelioma, have diagnosis codes which are categorized as benign in ICD‐O‐3 categorization of malignancies. These types (Kaposiform hemangioendothelioma, papillary intralymphatic angioendothelioma, retiform hemangioendothelioma, pseudomyogenic hemangioendothelioma) were thus not recorded in USCS or possible to include in our analyses. Although there have been improvements in survival of patients with Kaposiform hemangioendothelioma in recent years due to sirolimus based regimens, we do not expect that would impact our findings.^17^ However, given the high predominance of *WWTR1‐CAMTA1* gene fusions in patients with hemangioendothelioma and the consistency in demographics and survival outcomes that we demonstrated between the separate EHE and malignant hemangioendothelioma groups, we believe that this data still reflects a useful comparator.

As noted in a recent EHE consensus paper,^8^ better survival estimates are needed to serve as a benchmark for future interventional trials. Knowing a benchmark is especially critical for this ultra‐rare sarcoma where randomized studies are not feasible even with multicenter collaboration. These data effectively represent the entire US population, capturing 99% of hemangioendothelioma diagnoses in the study period and can serve as a benchmark for future single‐arm interventional studies.

## AUTHOR CONTRIBUTIONS

**Kelly G. Paulson:** Conceptualization (equal); data curation (equal); formal analysis (equal); investigation (equal); methodology (equal); project administration (equal); writing – original draft (equal); writing – review and editing (equal). **Vinod Ravi:** Writing – review and editing (equal). **Brian P. Rubin:** Writing – review and editing (equal). **Min Park:** Writing – review and editing (equal). **Elizabeth T. Loggers:** Writing – review and editing (equal). **Lee D. Cranmer:** Writing – review and editing (equal). **Michael J. Wagner:** Conceptualization (equal); formal analysis (equal); investigation (equal); methodology (equal); project administration (equal); writing – original draft (equal); writing – review and editing (equal).

## FUNDING INFORMATION

Work supported in part through the Paul G. Allen Research Center at Swedish Cancer Institute (K.G.P.) and by support to the Sarcoma Oncology Program of the University of Washington from Curt and Elizabeth Anderson and P30CA015704 (E.T.L., L.D.C., M.J.W.). Dr. Cranmer was supported in part by the Curt and Elizabeth Anderson Endowed Professorship in Sarcoma Research, and by funding from Steve and Jane Urner.

## CONFLICT OF INTEREST STATEMENT

KGP, VR, MP, BPR—nothing to disclose. ETL—Clinical trial funding from BioAtla, Ayala, SpringWorks. LDC—Clinical trial funding from Eli Lilly, AADi, BluePrint Medicine, Iterion, Gradalis, Philogen, Advenchen Laboratories, and CBA Pharma. Honoraria or has served on advisory boards for Daaichi Sankyo, BluePrint Medicines and Regeneron. MJW—Clinical trial support from Deciphera, Adaptimmune, GSK, Athenex, Foghorn Therapeutics, Shaqsi, Presage Biosciences, Inhibrx, Incyte. Consulting fees from Adaptimmune, Epizyme, Aadi, Deciphera.

## Supporting information


Figure S1:

## Data Availability

All data are publicly available through the U.S. Cancer Statistics (https://www.cdc.gov/cancer/uscs/index.htm).
