# Supplementary material for: Incidence, demographics, and survival of malignant hemangioendothelioma in the United States
Source: Cancer Med. 2023 Jun 1;12(14):15101–6. doi: 10.1002/cam4.6181 (PMC10417180; doi:10.1002/cam4.6181)
Supplement: Supplementary file 1 — Figure S1: [file CAM4-12-15101-s001.docx]

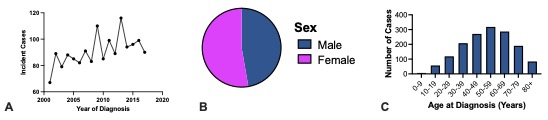
**Supplemental Figure 1:** Incidence and demographics of malignant hemangioendotheliomas characterized as EHE. The USCS data set only includes hemangioendotheliomas characterized as malignant per ICD-O-3 code. Of these the majority of reported cases are specified as epithelioid (n=1,542), which have similar demographics and outcomes to those classified as “malignant hemangioendothelioma” without further specification. Incident cases and demographics are shown for the EHE subset for reference. A) Incident cases – rate of EHE incidence is approximately 0.4 cases per million person-years, corresponding to ~100 EHE cases/year in US, B) sex, and C) age distribution of EHE. Survival outcomes for the EHE subset of malignant hemangioendothelioma are shown in Figure 3B.
